# Lactobacillus rhamnosus GG Outcompetes Enterococcus faecium via Mucus-Binding Pili: Evidence for a Novel and Heterospecific Probiotic Mechanism

**DOI:** 10.1128/AEM.01243-16

**Published:** 2016-09-16

**Authors:** Hanne L. P. Tytgat, François P. Douillard, Justus Reunanen, Pia Rasinkangas, Antoni P. A. Hendrickx, Pia K. Laine, Lars Paulin, Reetta Satokari, Willem M. de Vos

**Affiliations:** a Laboratory of Microbiology, Wageningen University, Wageningen, The Netherlands; b Department of Veterinary Biosciences, University of Helsinki, Helsinki, Finland; c Department of Medical Microbiology, University Medical Center Utrecht, Utrecht, The Netherlands; d Institute of Biotechnology, University of Helsinki, Helsinki, Finland; e RPU Immunobiology, Department of Bacteriology and Immunology, Haartman Institute, University of Helsinki, Helsinki, Finland; Pennsylvania State University

## Abstract

Vancomycin-resistant enterococci (VRE) have become a major nosocomial threat. Enterococcus faecium is of special concern, as it can easily acquire new antibiotic resistances and is an excellent colonizer of the human intestinal tract. Several clinical studies have explored the potential use of beneficial bacteria to weed out opportunistic pathogens. Specifically, the widely studied Lactobacillus rhamnosus strain GG has been applied successfully in the context of VRE infections. Here, we provide new insight into the molecular mechanism underlying the effects of this model probiotic on VRE decolonization. Both clinical VRE isolates and L. rhamnosus GG express pili on their cell walls, which are the key modulators of their highly efficient colonization of the intestinal mucosa. We found that one of the VRE pilus clusters shares considerable sequence similarity with the SpaCBA-SrtC1 pilus cluster of L. rhamnosus GG. Remarkable immunological and functional similarities were discovered between the mucus-binding pili of L. rhamnosus GG and those of the clinical E. faecium strain E1165, which was characterized at the genome level. Moreover, E. faecium strain E1165 bound efficiently to mucus, which may be prevented by the presence of the mucus-binding SpaC protein or antibodies against L. rhamnosus GG or SpaC. These results present experimental support for a novel probiotic mechanism, in which the mucus-binding pili of L. rhamnosus GG prevent the binding of a potential pathogen to the host. Hence, we provide a molecular basis for the further exploitation of L. rhamnosus GG and its pilins for prophylaxis and treatment of VRE infections.

**IMPORTANCE** Concern about vancomycin-resistant Enterococcus faecium causing nosocomial infections is rising globally. The arsenal of antibiotic strategies to treat these infections is nearly exhausted, and hence, new treatment strategies are urgently needed. Here, we provide molecular evidence to underpin reports of the successful clinical application of Lactobacillus rhamnosus GG in VRE decolonization strategies. Our results provide support for a new molecular mechanism, in which probiotics can perform competitive exclusion and possibly immune interaction. Moreover, we spur further exploration of the potential of intact L. rhamnosus GG and purified SpaC pilin as prophylactic and curative agents of the VRE carrier state.

## INTRODUCTION

For years, enterococci were viewed as harmless endogenous members of the human microbiota (1), with some strains even being used as probiotics (2). However, over 25 years ago, concerns started to grow as vancomycin-resistant enterococci (VRE) developed into a rapidly spreading nosocomial threat (3–6).

Presently, the prevalence of nosocomial infections caused by VRE is escalating globally (4, 5, 7, 8). These Enterococcus species are successful nosocomial agents since they are excellent colonizers, show striking genome plasticity, and persist for long periods of time in the intestinal tract, on medical devices, or on household appliances (4). Especially in immunosuppressed patients, VRE rapidly colonize the small intestine, cecum, and colon, thus outcompeting healthy gut microbiota. The virulence factors of VRE have yet to be fully unraveled, but the high colonization capacity of VRE is considered to be the main factor contributing to its pathogenicity (7, 9, 10). Moreover, VRE are remarkably efficient at acquiring antimicrobial resistances, further enhancing their persistence and colonization potential. The multiple antibiotic resistances of these enterococci are of special concern, as treatment of these infections becomes increasingly challenging. This further stimulates gut colonization by VRE, as the healthy gut microbiota is detrimentally affected during antibiotic treatment courses. The rapid acquisition of vancomycin resistance genes (*van* genes) is particularly worrisome (7, 8, 11). Vancomycin is the go-to antibiotic used to treat infections of Gram-positive bacteria that are known to be antibiotic resistant, and it is featured on the list of essential medicines of the WHO (12).

While initially the highly pathogenic Enterococcus faecalis was viewed as the major threat, it has been surpassed slowly but surely by its less pathogenic relative Enterococcus faecium. This trend is especially alarming, as E. faecium is highly efficient in the acquisition and accumulation of antibiotic resistance genes (4, 8, 10, 11).

To win the arms race between this bacterium and the search for new antibiotics, the need to evaluate alternative strategies is high. Several experts point out that the high colonization capability of E. faecium is the key feature to tackle (7, 9, 13). A plethora of strategies has been proposed, including “using the good to weed out the bad,” i.e., the administration of probiotics as a decolonization strategy (13, 14). In this respect, the capacity of Lactobacillus rhamnosus GG to eradicate VRE carriage has been evaluated in multiple clinical trials (13, 15–17). Administration of yogurt containing L. rhamnosus GG to renal patients resulted in the clearance of VRE in the treatment group, with most patients remaining VRE negative for at least 4 weeks after trial completion (16). In a Polish trial focusing on VRE-colonized children, L. rhamnosus GG treatment resulted in the temporary elimination of the VRE carrier state (17). In another clinical trial, focusing on adults with comorbidities, L. rhamnosus GG seemed to have no effect on VRE colonization, which may be due to the comorbidities presented by the patients and the short administration period (15). Based on these clinical trails and the revision of new case studies, Cheng and coworkers have suggested that a combination of antimicrobial therapy (including patient isolation and disinfection) and probiotic treatment with L. rhamnosus GG may hold the key for successful sustained decolonization of gastrointestinal carriage of VRE (13).

In this work, we provide new molecular insight into the mechanism underlying the role of L. rhamnosus GG in consolidation of VRE decolonization. Various Gram-positive species, including some lactobacilli, harbor pilus gene clusters that may be expressed as proteinaceous cell wall appendages, which contribute to their adhesion and signaling capacities (18–20). L. rhamnosus GG produces sortase-dependent pili that are decorated with the mucus-binding protein SpaC, to which its colonization capacities are attributed (21–24). Earlier work reported the enrichment of intact pilus gene clusters (containing LPXTG-type pilin proteins and a cognate class C-type sortase) in hospital-acquired E. faecium isolates (25, 26). We determined the complete genome sequence of the clinical E. faecium strain E1165. Comparison of pilin gene cluster 3 (PGC-3) of E. faecium E1165 and the SpaCBA pili of L. rhamnosus GG revealed a high level of sequence similarity between the two pilus gene clusters. Hence, we tested the capacity of the L. rhamnosus GG pili, and in particular the adhesive SpaC subunit, as well as antibodies raised against these components to interfere with E. faecium E1165 and its mucus interaction. Our findings not only demonstrate the existence of a novel immunogenic probiotic mechanism in the widely exploited intestinal isolate L. rhamnosus GG, but they also hold great promise for the development of prophylactic treatments.

## MATERIALS AND METHODS

### Bacterial strains and culture conditions.

Lactobacillus rhamnosus GG and its pilus-deficient derivative strain PB12 were grown at 37°C in MRS broth (Difco) in nonshaking conditions. Enterococcus faecium was grown stationary in brain heart infusion (BHI) broth (Difco) at 37°C. The three strains were grown in semianaerobic conditions, i.e., stationary growth and minimal oxygen. Escherichia coli was cultured aerobically at 37°C in LB medium under shaking conditions.

### Genome sequencing.

The genomic DNA of E. faecium isolate E1165 was extracted using the Wizard genomic DNA purification kit (Promega) per the manufacturer's recommendations. Genomic DNA sequencing of E. faecium isolate E1165 was performed using the Pacific Biosciences sequencing technology of the DNA Sequencing and Genomics Laboratory of the Institute of Biotechnology, University of Helsinki. In short, the PacBio library kit was used to construct a library with size targeted to 10 kb. Two single-molecule real-time (SMRT) cells were run on a PacBio RS II system using P6/C4 chemistry and 240 min of video time. A total of 78,536 reads (*N*_50_ length, 13,229 bp) were assembled using the HGAP.3 implemented in the SMRT analysis system (smrtanalysis_2.0.3; Pacific Biosciences). Apart from the complete chromosome (2,766,743 bp), a megaplasmid of 192,533 bp and one smaller plasmid of 32,402 bp were obtained with mean coverages of 190×, 210×, and 260×, respectively. Additionally, a circular phage genome (38,742 bp, 50×) was found. Using the RAST server (27), 3,024 open reading frames (ORFs) were predicted in the E. faecium E1165 genome and phage.

### Production of antiserum.

Serum samples were obtained from four patients that had E. faecium bacteremia. Samples were obtained during normal blood withdrawal and were approved by the medical ethical committee of the University Medical Center of Utrecht. PilB-specific rabbit immune serum and SpaC antiserum were produced as described earlier (25, 28). Antiserum against L. rhamnosus GG was produced as described recently (29). Controls with preimmune sera of PilB, SpaC, and L. rhamnosus GG were performed earlier and rendered negative results (25) (results not shown).

### Production of recombinant SpaC protein in E. coli.

The coding sequence of *spaC* (*LGG_00444*), excluding the C-terminal cell wall sorting signal and the N-terminal signal peptide, was PCR amplified from a codon-optimized *spaCBA* gene cluster (primers BG3764 ttcTCATGActgataacattcgcccaacc and BG3765 ttcGCGGCCGCcggcaaaattgcaagtgg). The purified PCR product was digested with BspHI and NotI (marked in capital letters in the primer sequences) and cloned into the pWUR533 vector, resulting in pWUR533_SpaC. This vector is a derivative of the pET52-1b vector in which the C-terminal thrombin protease site was replaced by an HRV 3C protease site and a Strep-tag II instead of a His tag. One percent of an overnight culture of E. coli BL21(DE3)/pSJS1244, in which the pWUR533_SpaC vector was introduced, was grown in LB medium with ampicillin (100 μg/ml) and spectinomycin (50 μg/ml). After 3 h of incubation at 37°C, protein production was induced by isopropyl-β-d-thiogalactopyranoside (IPTG) (1 mM). Three hours later, cells were harvested by centrifugation. Purification of the recombinant SpaC protein was performed in accordance with the Strep-Tactin resin manufacturer's recommendations (IBA). The Strep-tag was removed using the HRV 3C protease (EMD Millipore) following the protocol of the manufacturer. The tag and protease were subsequently removed using a Vivaspin 20 centrifugal concentrator (Sartorius).

### Mucus-binding assays.

Assessment of the binding of strains to mucus was performed as described earlier (30, 31). Bacterial cells were grown overnight in an appropriate medium supplemented with 1 mCi/ml [^3^H]thymidine (Perkin-Elmer Life Sciences). Bacteria (optical density at 600 nm [OD_600_], 0.25) were applied to a Nunc MaxiSorp plate coated with 0.5 mg/ml type II porcine mucus (Sigma-Aldrich, MO, USA). Optionally, 1/100 dilutions of antisera against total L. rhamnosus GG cells (raised as described in reference 29) and the SpaC subunit of the pili of L. rhamnosus GG (28) were added immediately to allow competition. Recombinant SpaC was added in a final concentration of 4.6 ng/μl. Bacteria were allowed to bind to mucus for 1 h at 37°C. After this, the plate was washed a few times with phosphate-buffered saline (PBS) prior to cell lysis (1 h at 60°C). The ^3^H measurement protocol has been extensively described previously (31).

### Immunogold electron microscopy.

Immunogold electron microscopy was performed as described earlier with minor modifications (21, 23, 25). In the first labeling step, the grids were incubated with rabbit SpaC antiserum diluted 1:120 in blocking solution or PilB antiserum diluted 1:5, followed by washing with 0.1% bovine serum albumin (BSA) in PBS. In the case of double-labeling experiments, a second labeling was performed using antisera in similar concentrations as described earlier. The grids were analyzed using a JEM-1400 transmission electron microscope (JEOL).

### Data analysis.

Data analysis was performed using GraphPad Prism 6. Significant differences between two values were calculated using paired *t* tests, and the significance level was set at a *P* value of <0.05.

### Accession number(s).

The complete circular genome, the two plasmid sequences, and phage sequences were deposited in the European Nucleotide Archive (ENA) under project ID PRJEB12401 and genome accession numbers LN999987 to LN999990.

## RESULTS

### Homology of pili of hospital-associated VRE and Lactobacillus rhamnosus GG.

To grasp the molecular basis of the decolonization effect of L. rhamnosus GG on VRE shown in clinical trials (13), we focused on their surface adhesion molecules. Specifically, SpaCBA pili of L. rhamnosus GG are described as primary adhesive molecules, which explains the mucus adhesion capacity of this probiotic (21, 22, 24, 31, 32). In addition, enterococci are known to produce pili that are important in colonization and adhesion to (a)biotic surfaces (19, 33, 34). The E. faecium type strain TX16 harbors four distinct pilin gene clusters (PGCs) (35), and hospital isolates of E. faecium are enriched in PGCs (25, 26).

**FIG 1:**
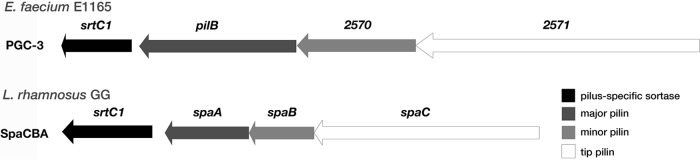
Sequence homology of pilus gene clusters of Enterococcus faecium E1165 and Lactobacillus rhamnosus GG. Pilus gene clusters of both E. faecium E1165 (∼99% similar to the TX16 type strain) (25) and L. rhamnosus GG (23) are shown. Overall, the clusters have a similar buildup and share a high sequence homology (>50% similarity), and both harbor a sortase (black), a tip adhesin (white), and two pilins (gray). The tip pilins of both strains ORF *2571* (TX16 and E1165) and *spaC* share 33% identity (49% positive matches). Arrow sizes reflect gene sizes.

A homology search exploring potential similarity between the PGCs of L. rhamnosus GG and E. faecium resulted in a striking similarity (>50%) between the SpaCBA-SrtC1 pilus cluster of L. rhamnosus GG and the PGC-3 cluster of E. faecium. Scanning the organization of the PGC-3 gene cluster, harboring the PilB-type pili (25), revealed a similar sequence and organization as the well-studied SpaCBA pili of L. rhamnosus GG (21, 22, 24, 36) (Fig. 1). Closer investigation revealed that the SpaC pilin of L. rhamnosus GG was 33% identical to the tip pilin protein sequence of the E. faecium TX16 strain (ORF *2571*). This similarity rose up to 49% when taking the amount of positive matches into account. Overall, mapping of the SpaC protein sequence to the E. faecium taxonomy identification number (taxid) 1352 revealed that in some strains, the sequence similarity even surpasses 50% (54% and up to 65% when considering positive matches) (data not shown).

As a case study for hospital acquired VRE, we focused on the clinically relevant E. faecium strain E1165, originally isolated from the wound/soft tissue of a hospitalized patient in 1997 (Genoa, Italy). Expression of the PGC-3 cluster-encoded PilB-type pili in E. faecium E1165 was already confirmed previously (25). We used PacBio RS II long-read sequencing technology to determine the complete genome sequence of strain E1165, which was deposited in the European Nucleotide Archive (ENA) under project ID PRJEB12401 and genome accession numbers LN999987 to LN999990. Detailed analysis of the E1165 genome revealed that the 2.766-Mb large genome contains three pilin gene clusters, as well as an additional pilin gene cluster (PilA gene cluster or PGC-1) located on a megaplasmid of 192.533 kb (37). The four gene clusters of E1165 are highly (∼99%) homologous to the PGCs of the TX16 type strain (data not shown).

### Cross-reactivity of antibodies reveals structural and immunological similarities between pili of E. faecium E1165 and L. rhamnosus GG.

**FIG 2:**
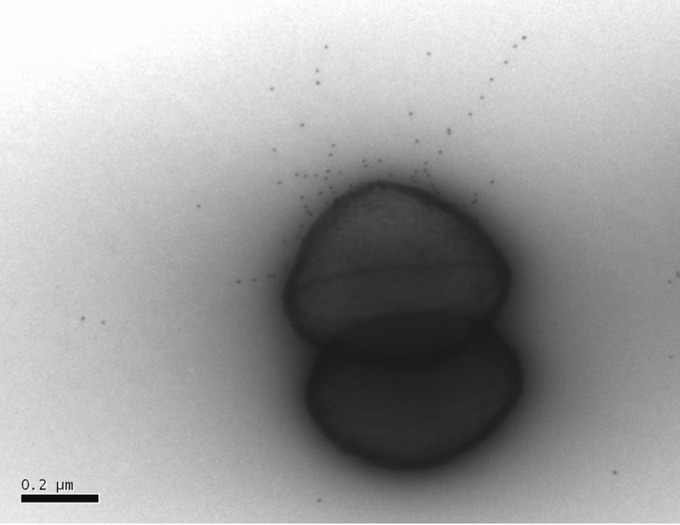
L. rhamnosus GG SpaC pilin-specific antibodies bind to E1165 pili. Immunogold labeling microscopy using SpaC antibodies shows binding of these L. rhamnosus GG major pilin-specific antibodies to the pili of E. faecium E1165 cells. Representative experimental results are shown (scale bar, 200 nm).

**FIG 3:**
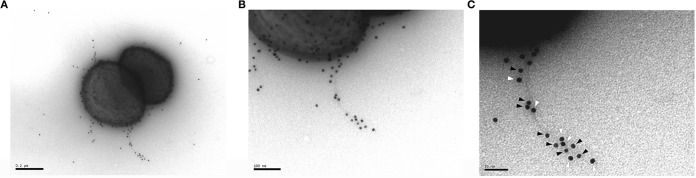
L. rhamnosus GG SpaC pilin antibodies colocalize with E. faecium PilB pilin antibodies on E1165 pili. E. faecium E1165 cells were incubated with gold particles either labeled with PilB antibodies (10-nm particles, black arrows in C) or SpaC antibodies (15-nm particles, white arrows in C). Both labels colocalize on the pili of E1165, indicating their specificity for the PilB-type pili of E. faecium E1165 expressed by PGC-3. (A) Complete E1165 cells (scale bar, 200 nm). (B and C) Zoomed-in image of the labeled pili (scale bars, respectively, 100 nm and 50 nm). Results of a representative experiment are shown.

To explore the functional relationship between the L. rhamnosus GG and E. faecium E1165 pili, we performed immunogold electron microscopy experiments using antibodies directed against the pilin subunits of each of these strains. Interestingly, antibodies raised against the SpaC subunit of the SpaCBA pili of L. rhamnosus GG specifically bound to the pili on the surface of E1165 (in immunogold labeling experiments) (Fig. 2). These results suggest that the sequence similarity between the SpaC pilin of L. rhamnosus GG and the tip pilin of E1165 may be indicative of a stronger, antigenic relation between the two pilus clusters. Next, we sought to investigate the nature of the targeted pili by setting up a double-labeling experiment that included both antibodies raised against the SpaC pilin of L. rhamnosus GG and PilB of E1165. Immunogold electron microscopy revealed that PilB is located in the same E. faecium pilus fiber that also reacts with gold-labeled antibodies against SpaC. This colocalization of both antibodies on the pili of E1165 confirms them being PilB-type pili expressed by PGC-3 (Fig. 3). This cross-reactivity of L. rhamnosus GG and E1165 pilin-specific antibodies illustrates that these pili are not only related on a genetic level but also on an antigenic level. The sequence homology seems to result in structurally related pilins, thus explaining the cross-reactivity of the antibodies.

**FIG 4:**
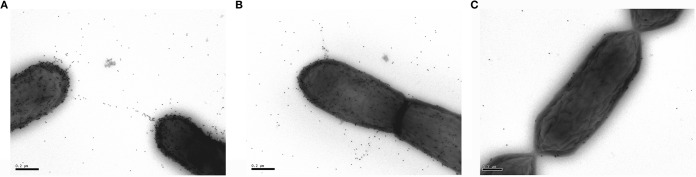
Serum from VRE-colonized patients interacts with L. rhamnosus GG pili, as shown by immunogold labeling microscopy of L. rhamnosus GG in patient serum samples. (A and B) Serum samples from two different patients infected with VRE. (C) Serum from a healthy patient (scale bar, 200 nm). Pictures of representative experiments are shown.

Next, we set out to relate these results to the clinical effect of L. rhamnosus GG administration on VRE colonization (13, 16, 17). Serum samples of patients with VRE bacteremia and healthy persons were isolated. Immunogold electron microscopy revealed that the serum samples of all VRE-colonized patients bound to L. rhamnosus GG, with the highest binding density on the SpaCBA pili (Fig. 4A and B). As expected, this interaction was absent when testing serum samples from healthy patients (Fig. 4C). These results suggest that the pili of both species are indeed related and that their sequence and antigenic homology might underpin the clinical results.

### L. rhamnosus GG-derived molecules effectively displace E. faecium from mucus.

Ultimately, we investigated whether the sequence and antigenic homology between the pili of L. rhamnosus GG and E1165 could be exploited in the decolonization of E. faecium as suggested by clinical trials (13). As pili are known to be key in the colonization and adhesion of microbes in the human intestinal tract by binding to human mucus (38, 39), we investigated the mucus-binding capacities of L. rhamnosus GG and E. faecium E1165 in the presence and absence of specific antibodies against the former.

**FIG 5:**
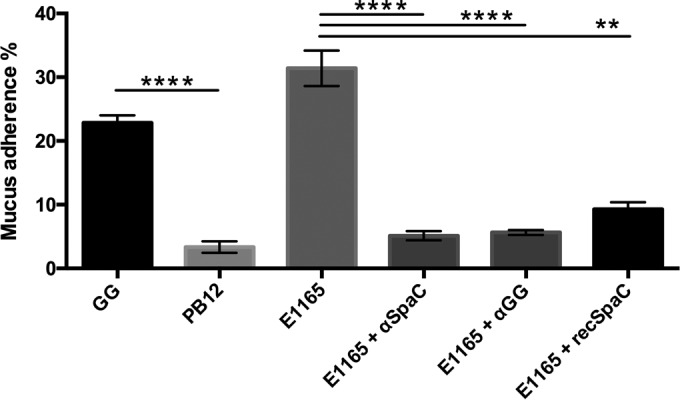
L. rhamnosus GG-specific antibodies and purified SpaC pilin interfere with the mucus-binding capacity of E. faecium E1165. The high adhesion capacity of L. rhamnosus GG to mucus can be explained by the presence of its SpaCBA pili, as adhesion of PB12, the mutant lacking pili, significantly drops (*P* < 0.0001). E. faecium E1165 can adhere well to mucus. Antibodies raised against the SpaC pilin of L. rhamnosus GG significantly reduce binding of E. faecium E1165 to mucus (*P* < 0.0001), as do antibodies raised against complete L. rhamnosus GG cells (*P* = 0.0004). The mucus-binding SpaC pilin purified from E. coli may also significantly interfere with binding of E1165 to mucus (*P* = 0.0011). Error bars depict the standard error of the mean (SEM) and data represent findings from at least triplicate experiments.

In accordance with earlier results, L. rhamnosus GG strongly bound to mucus (21, 24, 31). This interaction may be attributed largely to the presence of the SpaCBA pili, as a pilus-deficient derivative of L. rhamnosus GG, strain PB12, can only minimally adhere to mucus (31) (*P* < 0.0001) (Fig. 5). E. faecium E1165 was also found to interact strongly with the mucus (Fig. 5). Interaction levels were significantly higher (*P* < 0.05) for strain E1165 than those for L. rhamnosus GG, which may be indicative of a stronger binding of E1165 to mucus or the presence of more pili per cell. In view of the similarity between the pili of E. faecium and those of L. rhamnosus GG, we hypothesized that the E. faecium E1165 pili are the principal actors of its strong mucus adhesion. Hence, we sought to study the capacity of antibodies raised against L. rhamnosus GG and its SpaC pilin to affect E. faecium E1165 mucosal adhesion. Antibodies that were raised against the SpaC adhesive pilin effectively interacted with the binding of E1165 to mucus (Fig. 5). In addition, antibodies raised against intact L. rhamnosus GG cells could significantly interfere with E. faecium E1165 mucus binding (*P* < 0.0001) (Fig. 5). The effectiveness of the anti-L. rhamnosus GG antibodies can most probably be explained by the presence of reactive anti-SpaC antibodies in the polyclonal serum. Finally, the SpaC pilin produced and purified from E. coli was found to significantly reduce (*P* < 0.05) the mucus binding of E. faecium E1165 (and L. rhamnosus GG) (data not shown) (Fig. 5). In conclusion, these data indicate that L. rhamnosus GG and E. faecium E1165 pili compete for the same binding sites on mucus.

## DISCUSSION

Infections with vancomycin-resistant E. faecium or VRE have become a major nosocomial threat (4). Effective treatment can no longer rely on the application of antibiotics, as these bacteria are extremely efficient at accumulating antibiotic resistance genes in their genomes (11). This is related to the notion that multidrug-resistant enterococci lack the clustered regularly interspaced short palindromic repeat (CRISPR)-Cas system, a natural defense system (40). The full genome sequence of E. faecium strain E1165 did not reveal the presence of any CRISPR-Cas-related genes, reflecting its capacity to rapidly acquire antibiotic resistance genes (CRISPRdb screening [41]; results not shown).

New strategies to tackle VRE are currently being explored, including the implementation of probiotics such as L. rhamnosus GG (13). Several clinical trials have explored the efficacy of L. rhamnosus GG to interfere with VRE colonization in different setups (13, 15–17). Various studies, focusing on renal patients (16), children (17), and patients suffering from endocarditis and liver problems (13), illustrated the efficacy of L. rhamnosus GG administration in sustained colonization resistance against VRE. The effect of L. rhamnosus GG on VRE decolonization has been consistently demonstrated by all successful clinical trials. As the SpaCBA pili of L. rhamnosus GG are the main actors of its high adhesive phenotype, we sought to investigate the potential role of these molecules in its beneficial clinical effect against VRE colonization. Intriguingly, the SpaCBA pili of L. rhamnosus GG show a striking sequence homology to the PilB-type pili of enterococci encoded by the PGC-3 cluster. This is of special importance, as pilus gene clusters are enriched in pathogenic VRE strains (25, 26). A clinical isolate, E. faecium E1165, was earlier shown to express these PilB-type pili (25). The complete genome analysis of E1165 confirmed the presence of an intact PGC-3 cluster in its genome, and this strain was further used throughout the study.

Immunogold electron microscopy experiments revealed that the pili of L. rhamnosus GG and E. faecium E1165 not only share a high sequence homology but are also immunologically related. In particular, antibodies raised against the SpaC pilin of L. rhamnosus GG specifically interacted with the pili of E. faecium E1165. Further double-labeling experiments using specific antibodies against SpaC and PilB resulted in their colocalization on the PilB pili of E. faecium E1165. These results underline the antigenic relation between the two pilus clusters. Furthermore, we can relate these results to the clinical trials (13), as patient serum samples from VRE carriers interacted with L. rhamnosus GG cells and, in particular, with the surface-exposed and mucus-binding pili.

As colonization of the human intestine is most likely to rely predominantly on the interaction between bacteria, and more particularly on their adhesive pili, and the intestinal mucus (38, 39), the effect of L. rhamnosus GG on VRE mucus-binding was explored. These experiments resulted in the conclusion that antibodies raised against L. rhamnosus GG and its SpaC pilin interfered with the mucus-binding capacity of E. faecium E1165. The importance of the pili in this effect was further underpinned by the capacity of the purified SpaC pilin to drastically reduce the binding of E. faecium E1165 to mucus.

Based on our results, we suggest a novel probiotic mechanism in which probiotics are not only applied to perform interspecies competitive exclusion but are also inducers of cross-immunity against potential pathogens. The effect of L. rhamnosus GG on the decolonization of VRE implies a specific mechanistic effect of this probiotic on the gut community, which has not been reported to date.

In this work, we have successfully elucidated a molecular mechanism underlying the successful application of L. rhamnosus GG to eradicate E. faecium in several clinical trials and spur the further implementation of L. rhamnosus GG in VRE treatment (13). L. rhamnosus GG and its purified SpaC pilin can play important roles in the improvement of VRE treatment, the consolidation of its decolonization, and the development of effective strategies to tackle the (asymptomatic) carrier state of VRE. Our results shed light on a unique mechanism to specifically target VRE without harming commensal microbiota.
